# Are micro enemas administered with a squeeze tube and a 5 cm-long nozzle as good or better than micro enemas administered with a 10 cm-long catheter attached to a syringe in people with a recent spinal cord injury? A non-inferiority, crossover randomised controlled trial

**DOI:** 10.1038/s41393-022-00835-5

**Published:** 2022-07-27

**Authors:** Louise C. Kelly, Joanne V. Glinsky, Lianne M. Nier, Gillian Garrett, Lisa A. Harvey

**Affiliations:** 1 Royal North Shore Hospital, St Leonards, NSW Australia; 2 John Walsh Centre for Rehabilitation Research, Faculty of Medicine & Health, University of Sydney, Sydney, NSW Australia; 3 Northern Sydney Local Health District, Sydney, NSW Australia; 4 Royal Rehab, Ryde, NSW Australia

**Keywords:** Randomized controlled trials

## Abstract

**Study design:**

Double blind, non-inferiority crossover randomised controlled trial.

**Objectives:**

To determine if micro enemas administered with a squeeze-tube and a 5 cm-long nozzle (squeeze-tube method) are as good or better than micro enemas administered with a 10 cm-long catheter attached to a syringe (catheter method) in people with a recent spinal cord injury.

**Setting:**

Two inpatient spinal cord injury units located in Sydney, Australia.

**Methods:**

Twenty people admitted to hospital with recent spinal cord injury were randomly assigned to two treatment sequences; 4 weeks of micro enemas delivered by the squeeze-tube method followed by 4 weeks of micro enemas delivered by the catheter method, or vice versa. Each treatment sequence was 8 weeks with a crossover at the end of week 4. The primary outcome was time to complete bowel care. Secondary outcomes reflected faecal incontinence, quality of life, perception of treatment effectiveness and participant reported time to complete bowel care. The primary and secondary outcomes were measured by blinded assessors in week 4 and week 8. A non-inferiority margin of 10 min for time to complete bowel care was set a priori.

**Results:**

The mean between group difference (95% confidence interval) for the time to complete bowel care was −0.5 min (−2.8 to 1.8), where a negative value favours the catheter method. Results were similar for all secondary outcomes.

**Conclusions:**

Micro enemas delivered by the squeeze-tube method are as good or better than micro enemas delivered by the catheter method in people with a recent spinal cord injury.

## Introduction

**Fig. 1:**
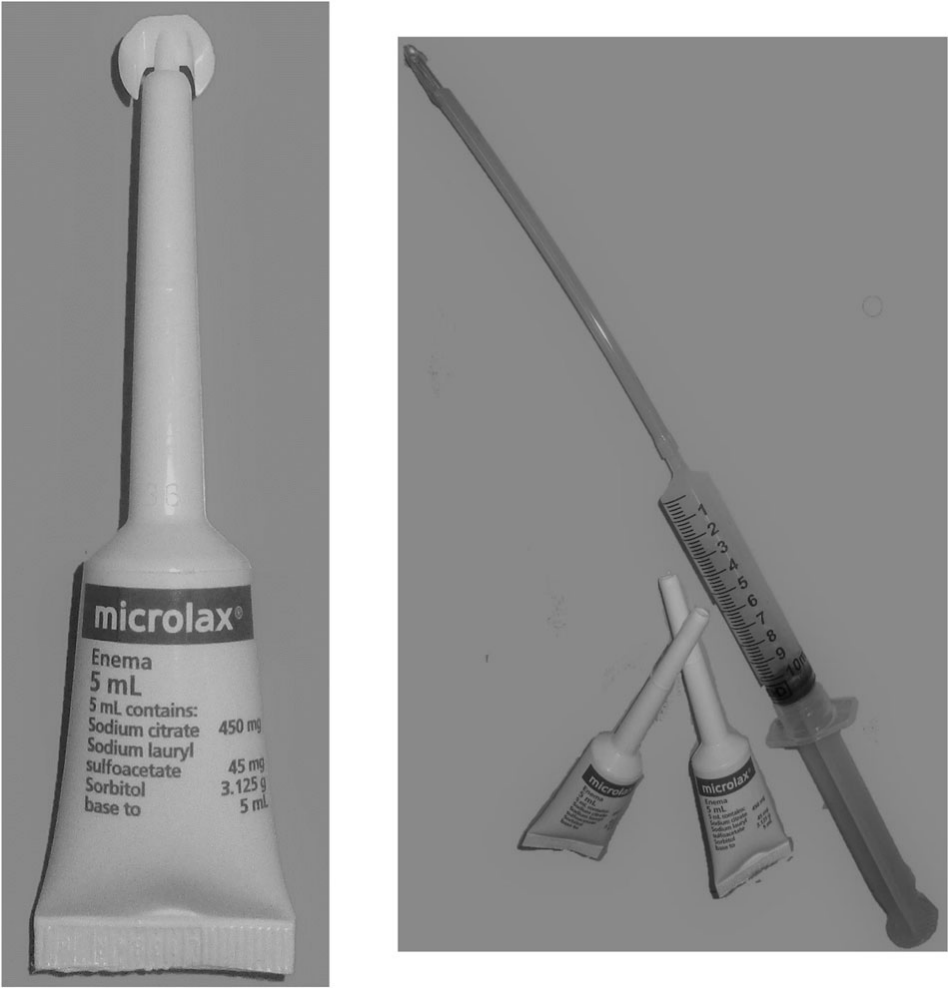
The squeeze-tube (left image) and catheter attached to a syringe (right image) methods of administering micro-enemas.

The managmement of neurogenic bowel dysfunction in people with spinal cord injury (SCI) can include the regular use of small volume enemas, called micro enemas [1–4]. These soften stools and draw water into them, to prompt bowel motions. They are preferable to high-volume enemas because they are less irritating on the rectum [4]. Micro enemas can be administered in one of two ways. The first and most comon way to administer a micro enema is with the commercially available squeeze tubes that have 5 cm-long nozzles that are inserted into the anal canal (not past the anorectal ring; see Fig. 1 left image). The second and less common way to administer micro enemas is with a syringe and 10 cm-long nelaton catheter (as used by females for clean intermittent catheterisation but shortened; (see Fig. 1 right image) [5]. The catheter gets the micro enema past the anorectal ring (but not past the rectal ampulla). The two ways of administering micro enemas are colloquially called “low” and “high” micro enemas but throughout this paper we will refer to the two methods as the “squeeze-tube” and the “catheter” methods to avoid confusion with the term, “high-volume enemas” [6, 7].

Very little research has been directed at the pros and cons of the squeeze-tube and catheter methods of administering micro enemas. There are obvious advantages of the squeeze-tube method. Namely, they are commercially available, easy to use and cheaper than syringes and catheters (assuming they are not reused). They may also cause less irritation to the rectum [8]. However, there is a strong belief (in Sydney, Australia at least) that the inconvenience and added cost of the catheter method is warranted because they decrease the time spent on toileting and are more effective. The beliefs are so strong that one Sydney SCI unit has been routinely using this method to administer micro enemas for at least 20 years. In the other Sydney SCI unit this method is also used but only if patients are constipated for several days. The different policy between the two Sydney SCI units creates complexity for community nurses who have to learn both methods of administering micro enemas and need to know which patient is using what. We therefore wanted to resolve the dilemma and determine whether the squeeze-tube method is as good (if not better) than the catheter method. It was decided that this should be based on time to complete bowel care as this was the most often argued justification for the use of the catheter method. A priori it was agreed that the squeeze-tube method would be deemed as good or better than the catheter method even if people with SCI spent up to 10 min longer toileting using the squeeze-tube method than the catheter method. The 10 min was nominated after consulting with 25 clinicians (doctors, nurses and surgical dressers (equivalent to nurse assistants)), 3 academics and 20 patients prior to the commencement of the trial. The possibility of an extra 10 min on toileting seemed a reasonable price to pay for the increased simplicity and reduced cost of the squeeze-tube method. The aim therefore of this study was to determine whether micro enemas delivered by the squeeze-tube method were as good (or better) than micro enemas delivered by the catheter method.

## Methods

### Design

A prospective, double-blinded, non-inferiority crossover randomised controlled trial was undertaken in two SCI units in Sydney, Australia. People admitted to the SCI units with a diagnosis of SCI were recruited into the trial from January 2019 until May 2021. Ethics approval was provided by the Northern Sydney Local Health District Human Research Committee. Written informed consent was obtained from all participants prior to enrolment into the trial. The trial was prospectively registered at the Australian New Zealand Clinical Trial Registry (www.anzctr.org.au; ACTRN12618000221257 with a Universal Trial Number: U1111-1194-6282). One important deviation from the registered protocol was that 20 participants (not 90 participants) were recruited to the trial. Recruitment had to cease between February 2020 and February 2021 due to the COVID-19 pandemic. As the study was being conducted as part of a PhD candidature, the trial was prematurely stopped once 20 participants had been recruited. The decision was made without looking at the data and solely for pragmatic reasons.

### Participants

All patients admitted to the SCI units of the Royal North Shore Hospital and Royal Rehab in Sydney, Australia were screened for trial eligibility and were considered for recruitment into the trial if they had a complete or incomplete SCI (as defined by the International Standards for Neurological classification of SCI) sustained within the past six months, had a return of peristalsis indicating a resolution of paralytic ileus, were stable on a bowel care routine, had an upper motor neuron lesion (as determined through intact anorectal reflexes and confirmed by rectal examination), were an inpatient at one of the participating SCI units and likely to remain there for the duration of the trial, were aged 16 years or over, and were willing to participate in the trial. Patients were excluded if they were unable to co-operate (e.g. a serious medical condition, cognitive impairment, drug dependency, psychiatric illness, or behavioural problem), were unable to speak sufficient English to provide consent and understand the study activities and assessments, were unable to tolerate a micro enema for medical reasons (i.e., contraindicated), had any type of neurological condition or injury other than a SCI affecting bowel function, had an allergy to any of the ingredients of a Microlax^®^ micro enema or had complications related to bowel care (e.g. haemodynamic instability or autonomic dysreflexia).

A secure random allocation schedule was computer generated prior to commencement of the trial by an independent person not involved in the recruitment of participants. Randomisation was stratified by site and stored in opaque, sequentially numbered and sealed envelopes kept at an off-site location. Once a participant was deemed suitable for the trial and baseline assessments were completed, an envelope was opened to reveal allocation. The participant was considered to have entered the trial at this point in time.

### Intervention

**Fig. 2:**
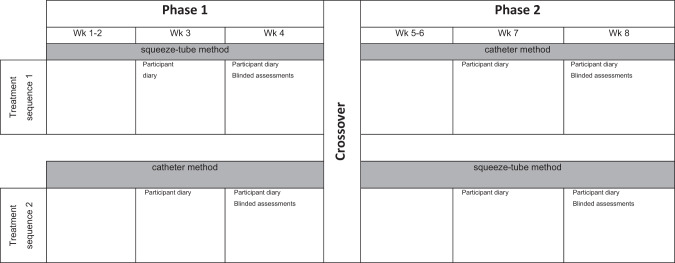
An overview of the design of the study indicating the two treatment sequences.

Each participant was on the trial for 8 weeks. Participants were assigned to either Treatment Sequence 1 or Treatment Sequence 2. Participants assigned to the Treatment Sequence 1 received daily micro enemas delivered by the squeeze-tube method for 4 weeks (phase 1) followed by daily micro enemas delivered by the catheter method for 4 weeks (phase 2). Participants assigned to the Treatment Sequence 2 received the reverse, namely daily micro enemas delivered by the catheter method for 4 weeks (phase 1) followed by daily micro enemas delivered by the squeeze-tube method for the next 4 weeks (phase 2) (Fig. 2). The initial 2 weeks of each 4-week phase was included as an active washout period because bowel care could not be withdrawn [9]. The additional two weeks was deemed sufficient to allow for adjustment to the new routine, given that any change to bowel routines take 3–4 bowel care cycles to take effect [10]. Participants in both Treatment Sequences continued to receive bowel care management by their health care team. This management included dietary advice, titration of aperients, provision of education and information regarding the effects of SCI on bowels and general bowel care troubleshooting. This management was largely the same across both groups with small, short term variations to diet or aperients made when required.

One Microlax^®^ micro enema consisting of 5 mL of sodium citrate dihydrate, sodium lauryl sulfoacetate and sorbitol was administered for both methods (supplier: *Johnson & Johnson, New Brunswick, NJ, USA*). However, an additional 5 mL micro enema was used to prime the catheter for the catheter method (excess liquid was disposed of to ensure the person only received 5 mL). The micro enemas were administered by nurses or surgical dressers experienced with administration of micro enemas. They also undertook additional instruction and training specific to the trial protocol. There were no delays between the initial digital check and the administration of the enema. Adherence to the bowel care regime was facilitated by the routines of the spinal units. For example, the methods of administering micro enemas each day were documented on the medication charts. The micro enema could only be dispensed each day once signed off by the Registered Nurse. In addition, the nursing staff or surgical dressers responsible for bowel care were required to record each day the method used to administer the micro enema in bowel care charts and medical notes. The recording of bowel care in this way is part of standard practice.

Attempts were made to blind the participants to the treatment sequence. For example, the kidney dish containing the squeeze-tube or syringe was covered and participants were asked to close their eyes or turn their heads when it was administered during bowel care each day. At the end of the trial participants were asked questions to gauge the success of these efforts to blind them.

### Outcome measures

An independent, person blinded to treatment sequence allocation assessed the participants on 3 separate occasions in week 4 (to reflect the first phase) and on another 3 occasions in week 8 (to reflect the second phase). The assessors were nursing staff who were trained in the assessments and did not provide direct care to participants (i.e., Clinical Nurse Consultants, Clinical Nurse Educators, Clinical Nurse Specialists). Again, the success of this blinding was gauged by asking the assessors a series of questions at the end of each assessment.

The assessments (including the questionnaires) were repeated 3 times at the end of each phase to increase the precision of each estimate. The assessments were always the same and captured the following outcomes:

#### Time to complete bowel care (assessor reported) [11, 12]

This was the primary outcome for the trial. It was defined as the time from commencing toileting (initial digital rectal check) to completion (faecal elimination as defined by an empty rectum on digital rectal check).

#### Frequency of unplanned bowel evacuations

This was measured using the Faecal Incontinence Scoring System (also known as the Douglas Wong Score) [13]. This is a 5-item self-report questionnaire capturing frequency of faecal incontinence. Scores range from 0 (nil incontinence) to 120 (severe faecal incontinence). Final scores are attained by taking the single highest score from questions 1–4 and adding this to the score from question 5.

#### The Spinal Cord Injury Quality of Life (SCI QoL)—Bowel Management Difficulties Short Form [14]

This a patient reported outcome measure that captures quality of life due to bowel management difficulties. Raw scores range from 9 (T-score conversion 39.2) to 45 (T-score conversion 76.3) with higher scores representing more bowel management difficulties (worse function) than lower scores. The raw scores were converted into T scores (range 39.2 to 76.3) as recommended by Tulsky et al. [15].

#### Perception of effectiveness of bowel care routine (participant reported)

This captures participants’ perceptions of the effectiveness of their bowel routines over the past 4 weeks. It was measured using a 11-point Likert Scale (0–10), anchored at each end from “extremely ineffective” to “extremely effective”, respectively.

#### Perception of effectiveness of bowel care routine (clinician reported)

This captures the treating clinicians’ perceptions of the effectiveness of participants’ bowel routines over the past 4 weeks. It was measured using a 11-point Likert Scale (0–10), anchored at each end from “extremely ineffective” to “extremely effective”, respectively.

The following additional outcome was also used but collected differently and separately to the above outcomes:

#### Time to complete bowel care (participant reported)

Participants were asked to keep a diary during weeks 3 and 4 (to reflect the first phase), and then again during week 7 and 8 (to reflect the second phase). They were asked to record the time taken to complete bowel care on 6 separate occasions (with assistance as required). Participants were asked to use a timer or the stopwatch function on their phones to record the time from commencement of bowel routine (initial digital check) to the completion of their bowel routines (final digital check) [11, 12].

Some additional descriptive data were collected from participants to capture which method of administering the micro enema they preferred and why. Specifically, at the end of the trial the participants were asked to compare the two methods of delivery on a 15-point Likert scale (−7 to 7), anchored at each end to indicate whether one method was “very much worse” or “very much better” than the other. Participants were also asked what they liked and disliked about each method.

### Sample size

A non-inferiority margin for the primary outcome (time to complete bowel care: assesor reported) was set at 10 min a priori. That is, it was decided that the squeeze-tube method would be considered non inferior to the catheter method (i.e., the squeeze-tube method would be considered as good as or better than the catheter method) provided time to complete bowel care was not more than 10 min longer for the squeeze-tube method than the catheter method.

It was initially estimated that a sample size of 90 people would be required to provide 90% power for the non-inferiority margin (delta) of 10 min (where a negative between-group difference favours the catheter method) assuming a standard deviation of 18 min and loss to follow-up of 15% [12]. The SD was estimated from data (unpublished) initially collected from 22 inpatients in February 2017 and a previous crossover trial using similar outcomes [12].

### Data analysis

**Fig. 3:**
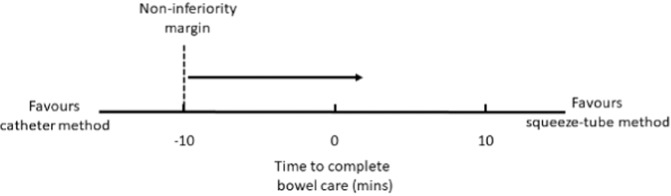
The interpretation of the non-inferiority margin. Micro enemas delivered with the squeeze-tube method would be deemed as good or better than micro enemas delivered with the catheter method provided the lower end of the 95% CI associated with the mean between-group difference did not span - 10 min (favouring the catheter method).

All data were entered into and managed using the Research Electronic Data Capture (REDCap) software hosted at the University of Sydney, Australia [16]. REDCap is a secure web-based software platform designed to support data capture for research studies. The data were analysed using intention to treat [17] and Stata software (Version 16, StataCorp LP, College Station, TX, USA). The analyses were conducted by LAH (who was blinded to group allocation) and independently confirmed by LCK. Mean values for the squeeze-tube and catheter methods were attained for each outcome from the repeated measurements taken at the end of each phase. Linear regression was used to determine the mean between-group differences (and 95% confidence intervals, CIs) for all outcomes. The 95% CI of the mean between-group difference of the primary outcome was interpreted with respect to the non-inferiority margin of 10 min as shown in Fig. 3. A post hoc sensitivity analysis (or per protocol analysis) was also conducted to determine the effect of the inclusion of two participants who did not adhere to the study protocol.

## Results

### Flow of participants through study

**Fig. 4:**
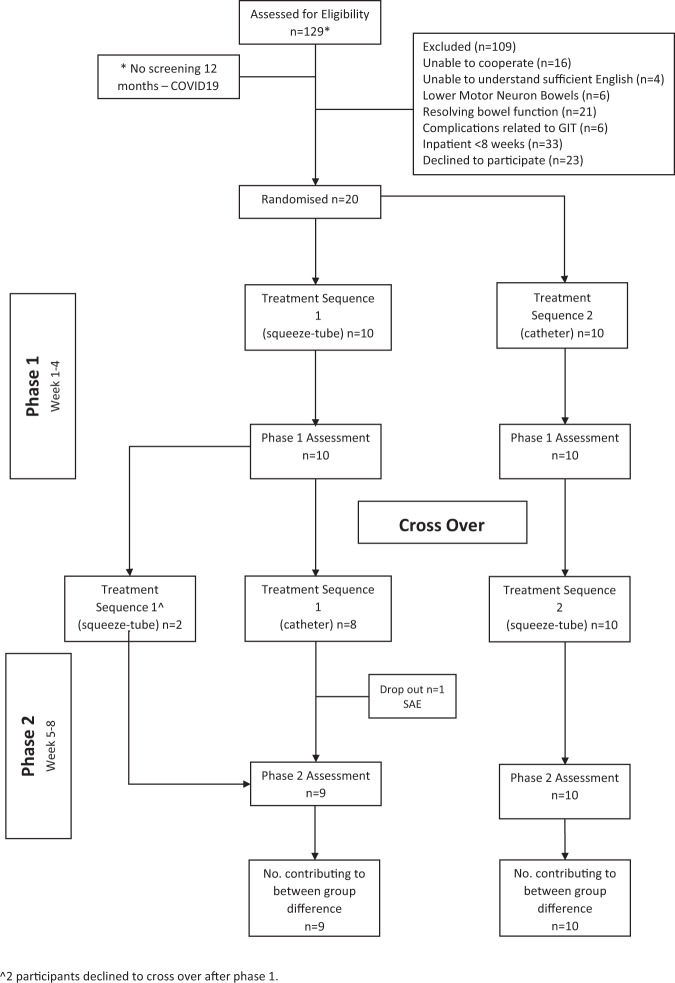
Flow of participants through the study.

A total of 129 people who had sustained a SCI and who were admitted to the Royal North Shore Hospital and Royal Rehab in Sydney were screened for eligibility to participate in the trial from January 2019 to May 2021 (excluding a 12-month period commencing early 2020 when the trial was ceased, and no screening logs were kept due to the COVID-19 pandemic). Twenty eligible participants were randomised. The flow of participants through the trial is shown in Fig. 4. Ten participants were allocated to Treatment Sequence 1 (squeeze-tube then syringe method) and ten to the Treatment Sequence 2 (syringe then squeeze-tube method). One participant dropped out of the trial at week 5 due to a serious adverse event (sub-dural haemorrhage and subsequent death). This was unrelated to the trial intervention. There were no other serious adverse events.

### Participant characteristics

Fifteen (80%) of the 20 participants were male, 17 (85%) participants had tetraplegia and three participants had paraplegia (15%). Participants had AIS A (*n* = 7), AIS B (*n* = 4), AIS C (*n* = 5) and AIS D (*n* = 4) lesions as defined by the International Standards for Neurological Classification of Spinal Cord Injury. The median (interquartile range) age was 65.9 years (30.3 to 73.5) and the median (interquartile range) time since injury was 1.5 months (0.8 to 2.0). The majority of participants (60%) had moderate bowel dysfunction as indicated by a median (interquartile range) score of 10 (5 to 11) on the Neurogenic Bowel Dysfunction Score (Table 1). The groups were similar at baseline on all demographic characteristics except age (a chance difference due to the small sample size).

**Table 1:** Participants’ demographic characteristics at baseline.

	Treatment sequence 1 *n* = 10	Treatment sequence 2 *n* = 10
Gender (M:F), *n*	6:4	9:1
Age (years), median (IQR)	39.5 (30.3–73.5)	67.3 (45.8–72.0)
Type of injury, *n*		
Traumatic	10	9
Non-traumatic	—	1
Time since injury (months), median (IQR)	1.5 (0.9–2.0)	1.4 (0.8–2.0)
Neurological level, *n*		
C2–C5	7	6
C6–C8	2	2
Thoracic	1	1
Lumbar	—	1
AIS classification, *n*		
A	3	4
B	4	—
C	1	4
D	2	2
Neurogenic Bowel Dysfunction Score (points/47)		
median (IQR)	10.0 (10.0–11.0)	7.5 (3.0–10.0)
Severity of dysfunction, *n*		
Score 0–6: very minor	2	5
Score 7–9: minor	—	—
Score 10–14: moderate	7	5
Score ≥14: severe	1	—

### Compliance with trial protocol

Two participants did not fully comply with the trial protocol. Both declined to crossover from the squeeze-tube to the catheter method after phase 1. However, all outcome measures were collected for these two participants and their data were analysed in accordance with the principles of intention to treat. Medication charts, medical records and bowel charts were used to verify that the other 17 participants received daily micro enemas with the appropriate method as per the protocol. This was largely the case except for one participant from Treatment Sequence 2 who had spontaneous bowel results prior to the administration of his micro enema on 5 occasions in phase 2. Another participant from Treatment Sequence 1 received a bisacodyl micro enema on three consecutive occasions due to constipation in phase 1, and one participant from Treatment Sequence 2 received a micro enema by the squeeze-tube method (rather than the catheter method) on two occasions at the beginning of phase 1.

All outcomes were collected at the designated times. The assessors reported that they remained blinded for all but one assessment when a syringe and catheter were left in a participant’s bathroom. The participants likewise reported to have remained blinded except for one who had anal sensation and could tell the difference between the two methods of delivery.

### Treatment effect

**Fig. 5:**
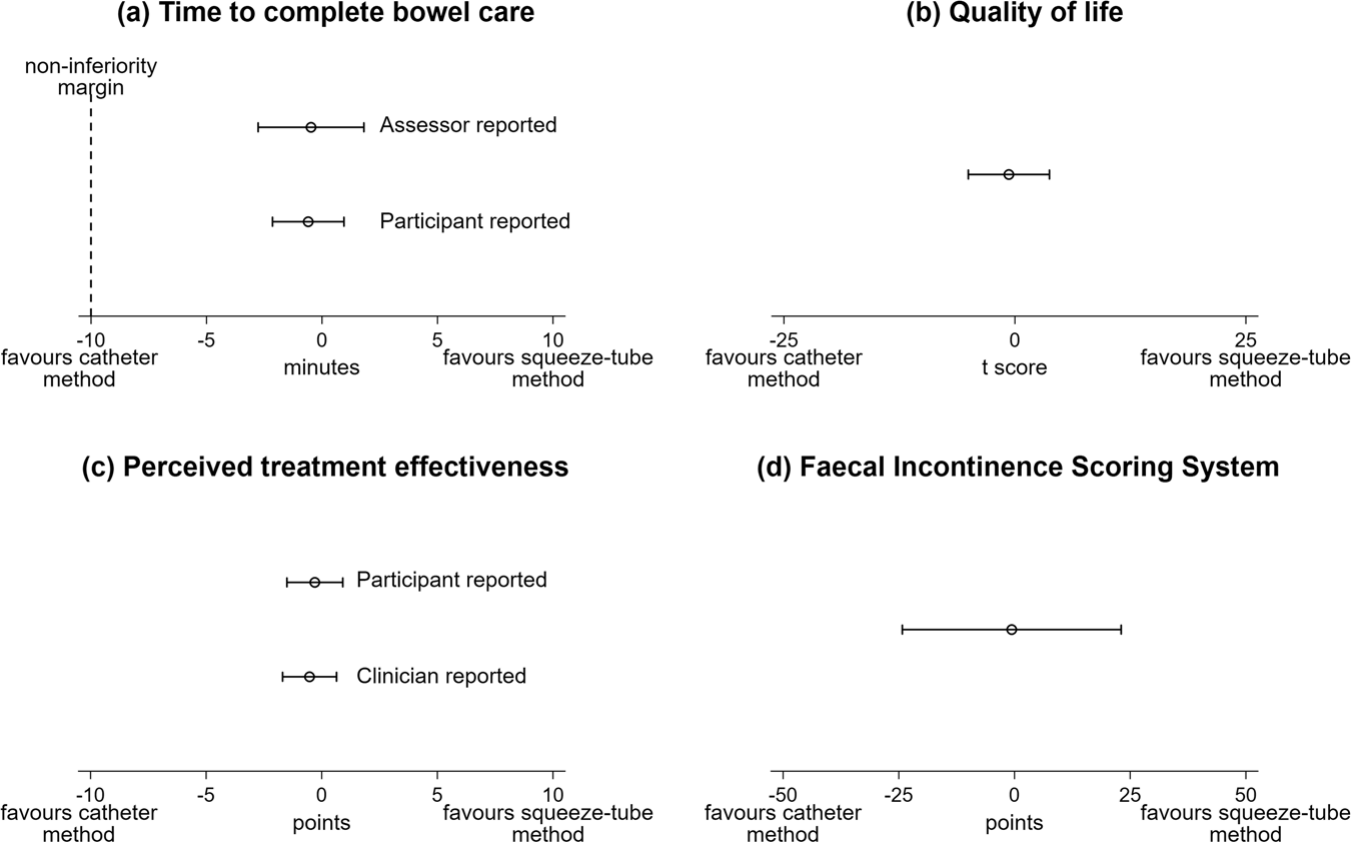
Mean between group difference (95% CI) for each outcome. **a** Time to complete bowel care. **b** Quality of life (as determined by the SCI-QOL Bowel Management Difficulties Score). **c** Perceived treatment effectiveness (participant and clinician reported). **d** Faecal incontinence score.

The results by the two phases and by methods of delivery are shown in Tables 2 and 3. The mean (95% CI) time to complete bowel care (assessor reported) was −0.5 mins (−2.8 to 1.8) favouring the catheter method (Fig. 5 and Table 3). As illustrated in Fig. 5, the lower limit of the 95% CI was within the non-inferiority margin of −10 min indicating that the squeeze-tube method is not inferior to the catheter method. These results were similar when the analyses were repeated with the removal of the two participants who did not adhere to the trial protocol (see the Supplementary File for details). The secondary outcome measures with the mean between group differences are presented in Table 3. All results support the interpretation of the analyses for the primary outcome. That is, the squeeze-tube method is not inferior to the catheter method.

**Table 2:** Outcomes by phases.

	Phase one		Phase two	
	Treatment sequence 1 (squeeze-tube) *n* = 9	Treatment sequence 2 (catheter) *n* = 10	Treatment sequence 1 (catheter) *n* = 9	Treatment sequence 2 (squeeze-tube) *n* = 10
	${{{\bar{\boldsymbol x}}}}$ (SD)	${{{\bar{\boldsymbol x}}}}$ (SD)	${{{\bar{\boldsymbol x}}}}$ (SD)	${{{\bar{\boldsymbol x}}}}$ (SD)
Time to complete bowel care (assessor reported) (mins)^a^	18.9 (6.5)	17.9 (7.5)	17.2 (4.0)	17.3 (5.4)
Faecal incontinence score system (points/0–120)^a^	38.6 (32.6)	51.8 (29.4)	37.7 (35.8)	52.1 (39.9)
SCI-QOL Bowel Management Difficulties T score (39.2–76.3)^a^	53.9 (4.9)	51.5 (6.9)	53.2 (8.2)	52.1 (9.2)
Perception of effectiveness of bowel care routine (participant reported) (points/0–10)^b^	7.7 (1.8)	8.0 (1.4)	7.5 (1.8)	7.3 (2.6)
Perception of effectiveness of bowel care routine (clinician reported) (points/0–10)^b^	7.8 (1.3)	7.6 (1.8)	7.6 (1.1)	6.4 (2.4)
Time to complete bowel care (participant reported) (mins)^a^	18.7 (5.6)	17.1 (6.2)	17.4 (4.5)	17.1 (5.7)

^a^A lower score indicates a better outcome.

^b^A higher score indicates a better outcome.

**Table 3:** Outcomes by the two methods of delivering the micro enemas with mean between group difference (95% CI).

	The squeeze-tube method *n* = 19 ${{{\bar{\boldsymbol x}}}}$ (SD)	The catheter method *n* = 19 ${{{\bar{\boldsymbol x}}}}$ (SD)	Mean between group difference (95% CI) *n* = 19
Time to complete bowel care (assessor reported) (mins)^a^	18.1 (5.8)	17.6 (5.9)	−0.5 (−2.8 to 1.8)
Faecal incontinence score system (points/0–120)^a^	45.7 (36.2)	45.1 (32.5)	−0.6 (−24.2 to 23.0)
SCI-QOL Bowel Management Difficulties T score (39.2–76.3)^a^	52.9 (7.3)	52.3 (7.4)	−0.7 (−5.0 to 3.7)
Perception of effectiveness of bowel care routine (participant reported) (points/0–10)^b^	7.5 (2.2)	7.8 (1.6)	−0.3 (−1.5 to 0.9)
Perception of effectiveness of bowel care routine (clinician reported) (points/0–10)^b^	7.1 (2.0)	7.6 (1.5)	−0.5 (−1.7 to 0.6)
Time to complete bowel care (participant reported) (mins)^a^	17.9 (5.6)	17.3 (5.3)	−0.6 (−2.1 to 1.0)

^a^A lower score indicates a better outcome, and a negative between-group difference favours the catheter method.

^b^A higher score indicates a better outcome, and a negative between-group difference favours the catheter method.

Whilst blinded, participants were asked to provide general information about their perceptions of the two methods of micro enema delivery. Comments and preferences were linked to the treatment sequence allocation during data analysis. The median (IQR) score reflecting which method participants preferred was 0.0 (−2.0 to 0.0) where −7 (very much worse) indicates a preference for the squeeze-tube method and +7 (very much better) indicates a preference for the catheter method. Participants expressed varying and often contradictory opinions when asked why they preferred one type of method over the other. For example, while one participant stated that his/her bowel care was 6 min quicker with the squeeze-tube method than the catheter method, others stated that the catheter method gave a quicker and more consistent result than the squeeze-tube method. Similarly, 3 participants reported decreased bowel accidents with the catheter method with one participant stating that he/she felt less anxious about having bowel accidents with the catheter method than the squeeze-tube method, yet others reported the opposite, namely increased bowel accidents and more unpredictable bowel results with the catheter method compared to the squeeze-tube method. One participant stated that the squeeze-tube method felt “hard” and another merely stated that he/she “didn’t like this [the catheter method of delivering the] enema.”

## Discussion

This is the first randomised controlled trial to compare the effects of two different ways of delivering micro enemas in people with recent SCI. The study is important for people with SCI because of the detrimental impacts on their lives of excessive time spent toileting, chronic constipation and faecal incontinence. Furthermore, the trial addresses a question that was posed by nursing clinicians in Sydney SCI units about which method of delivering micro enema should be routinely used for people with SCI. We have been able to add objective evidence to guide decisions around this question. The results of the trial clearly indicate that the less expensive and more convenient squeeze-tube method is as good if not better than the catheter method of delivering micro enemas for people with SCI and this interpretation is further supported by the results of all the secondary outcomes.

The interpretation of the results relies on accepting the non-inferiority margin of 10 min for the primary outcome. We selected this a priori after discussions with patients, researchers and clinicians. It was also based on initial data suggesting that people with SCI typically take 30 to 60 min to complete their bowel care [18–20]. However, this latter assumption proved to be incorrect. The mean time to complete bowel care in participants of this trial was only 18 min. It is not clear why time to complete bowel care in the participants of this trial was so much shorter than the anecdotal observations of the clinicians and the results of previous recent studies, however, this may reflect our inclusion of people soon after injury. There is some evidence to suggest that time to complete bowel increases with time post injury [21–26].

The results and interpretation of our primary outcome are supported by our secondary outcomes even though we did not set non-inferiority margins for each secondary outcome. Nonetheless, the lower end of the 95% CI associated with the mean between group differences for each secondary outcome indicates that the squeeze-tube method is as good as the catheter method with respect to faecal incontinence, quality of life and perceptions of effectiveness. In addition, the results for participant-reported time to complete bowel care nearly perfectly mirrored the same outcome as measured by the blinded assessors. Together these results increase our confidence in the main finding of this trial.

We chose a crossover design for several reasons. Firstly, in this trial, it enabled participants to experience both methods of delivery of the micro enemas. This allowed us to explore their responses and preferences [27]. Secondly, a crossover design increases the precision of estimates for the same sample size [28]. Our estimates were very precise (as reflected in the narrow 95% CI of the mean between-group differences) except for the Faecal Incontinence Score. We chose the Faecal Incontinence Score as it asked participants to reflect on faecal incontinence over the past 4 weeks. Most of the similar and commonly used outcome measures for people with SCI ask participants to reflect on the past 3 months [13, 29–33]. This is problematic for a trial like this. The disadvantage of the crossover design was the possibility of carry-over and period effects [9]. Carry-over effects can occur if the first method of delivery (i.e., the squeeze-tube or catheter method) influences the second method of delivery (i.e., syringe or squeeze-tube method). We attempted to guard against this by using an active washout period of 2 weeks (this is deemed sufficient by others) [10]. Period effects can occur if there is a tendency for time to complete bowel care to change merely with the passage of time. Whilst there are statistical methods to adjust for the possibility of both carry-over and period effects, we followed the guidance of Senn [9] and relied on the randomisation and design of the trial to minimise both [9, 27].

There are two notable weaknesses of our trial. Firstly, we planned to recruit 90 participants but had to end the trial early after 20. As it turned out this was a sufficient sample size to answer our research question. This can be explained by the smaller SD (5.9, Table 3) than anticipated (18, [12]). We based our SDs on the best available data but with little prior research in this area there was always a risk of error. We were fortunate that the error was in the right direction. The second weakness of our trial was related to the poor adherence to the protocol of two participants and the withdrawal of a third participant. These issues introduce bias to any trial but particularly to non-inferiority and crossover trials [28, 34, 35]. Nonetheless, our post hoc sensitivity analyses suggest that the results were reasonably robust to the inclusion of the non-compliant participants’ data (Supplementary File 1). That is, the results were almost identical with and without the data of these participants.

## Conclusion

In all, our results indicate that delivering micro enemas with the squeeze tube method is as good or better than delivering micro enemas with the catheter method provided patients and clinicians are willing to spend up to 2 min longer toileting. This possible slight increase in toileting time with the squeeze-tube method can be justified to save the additional expense and complexity associated with the catheter method. This trial addresses a very practical question about bowel care: a much under-researched area of care for people with SCI despite the clear importance to them.

## Supplementary information

Supplementary File

## Data Availability

The authors will consider reasonable requests for the participant level data.

## References

[1] Middleton JW, Arora M, McCormack M, O’Leary D. Health maintenance tool. How to stay healthy and well with a spinal cord injury. A tool for consumers by consumers. 1st ed. Sydney: Royal Rehab; 2020. https://www.icare.nsw.gov.au/-/media/icare/unique-media/global-header/news-and-stories/news/health-maintenance-toolkit-a-guide-for-people-with-spinal-cord-injury/spinal-cord-injury-health-maintenance-toolkit.pdf.

[2] Consortium for Spinal Cord Medicine. Management of neurogenic bowel dysfunction in adults after spinal cord injury. Clinical practice guideline for health care providers. Washington, DC: Paralyzed Vetrans of America; 2020. 10.46292/sci2702-75 PMC8152174 34108835

[3] Coggrave M, Norton C, Cody JD. Management of faecal incontinence and constipation in adults with central neurological diseases. Cochrane Database Systematic Rev. 2014;CD002115. 10.1002/14651858.CD002115.pub5 10.1002/14651858.CD002115.pub5 PMC10656572 24420006

[4] Multidisciplinary Association of Spinal Cord Injured Professionals. Guidelines for management of neurogenic bowel dysfunction inindividuals with central neurological conditions. London: Multidisciplinary Association of Spinal Cord Injured Professionals; 2012.

[5] The Spinalis Foundation. Customized syringe for microlax. Stockholm: Spinalistips; 2007. Accessed 22 Oct 2021. https://spinalistips.se/en/tip-customized-syringe-for-microlax-632

[6] Schmelzer M, Wright K (1996). Enema administration techniques used by experienced registration nurses. Gastroenterol Nurs..

[7] Doyle D (2005). Per rectum: A history of enemata. J R Coll Physicians Edinb.

[8] Johns JS, Krogh K, Ethans K, Chi J, Queree M, Eng JJ (2021). Pharmacological management of neurogenic bowel dysfunction after spinal cord injury and multiple sclerosis: A systematic review and clinical implications. J. Clin. Med..

[9] Senn S. Cross-over trials in clinical research, 2nd ed. J. Wiley: Chichester, Eng.; 2002: 10.1002/0470854596.

[10] Pryor J, Fisher M, Middleton J. Management of the neurogenic bowel for adults with spinal cord injurie*s*. NSW Health: North Sydney; 2014.

[11] Coggrave MJ, Norton C (2010). The need for manual evacuation and oral laxatives in the management of neurogenic bowel dysfunction after spinal cord injury: A randomized controlled trial of a stepwise protocol. Spinal Cord.

[12] Kwok S, Harvey L, Glinsky J, Bowden JL, Coggrave M, Tussler D (2015). Does regular standing improve bowel function in people with spinal cord injury? A randomised crossover trial. Spinal Cord.

[13] Wong DW, Congliosi SM, Spencer MP, Corman ML, Tan P, Opelka FG (2002). The safety and efficacy of the artificial bowel sphincter for fecal incontinence: Results from a multicenter cohort study. Dis Colon Rectum.

[14] Tulsky DS, Kisala PA, Victorson D, Tate DG, Heinemann AW, Charlifue S (2015). Overview of the spinal cord injury-quality of life (SCI-QoL) measurement system. J Spinal Cord Med.

[15] Tulsky DS, Kisala PA, Tate DG, Spungen AM, Kirshblum SC (2015). Development and psychometric characteristics of the SCI-QoL bladder management difficulties and bowel management difficulties item banks and short forms and the SCI-QoL bladder complications scale. J Spinal Cord Med..

[16] Harris PA, Taylor R, Thielke R, Payne J, Gonzalez N, Conde JG (2009). Research electronic data capture (REDcap)—a metadata-driven methodology and workflow process for providing translational research informatics support. J Biomed Inf..

[17] Pocock SJ. *C*linical trials: A practical apporach. Wiley: Chichester; 1983: 10.1002/9781118793916.

[18] Adriaansen JJ, van Asbeck FW, van Kuppevelt D, Snoek GJ, Post MW (2015). Outcomes of neurogenic bowel management in individuals living with a spinal cord injury for at least 10 years. Arch Phys Med Rehabil.

[19] Coggrave M, Norton C, Wilson-Barnett J (2009). Management of neurogenic bowel dysfunction in the community after spinal cord injury: A postal survey in the United Kingdom. Spinal Cord.

[20] Kim JY, Koh ES, Leigh J, Shin HI (2012). Management of bowel dysfunction in the community after spinal cord injury: A postal survey in the Republic of Korea. Spinal Cord.

[21] Krassioukov A, Eng JJ, Claxton G, Sakakibara BM, Shum S (2010). Neurogenic bowel management after spinal cord injury: A systematic review of the evidence. Spinal Cord.

[22] Glickman S, Kamm MA (1996). Bowel dysfunction in spinal-cord-injury patients. Lancet.

[23] Liu CW, Huang CC, Chen CH, Yang YH, Chen TW, Huang MH (2010). Prediction of severe neurogenic bowel dysfunction in persons with spinal cord injury. Spinal Cord.

[24] Faaborg PM, Christensen P, Finnerup N, Laurberg S, Krogh K (2008). The pattern of colorectal dysfunction changes with time since spinal cord injury. Spinal Cord.

[25] Stone JM, Nino-Murcia M, Wolfe VA, Perkash I (1990). Chronic gastrointestinal problems in spinal cord injury patients: A prospective analysis. Am J Gastroenterol.

[26] Krogh K, Nielsen J, Djurhuus JC, Mosdal C, Sabroe S, Laurberg S, et al. Colorectal function in patients with spinal cord lesions. Dis Colon Rectum.1997;40:1233–9. 10.1007/BF02055170 9336119

[27] Hui D, Zhukovsky DS, Bruera E (2015). Which treatment is better? Ascertaining patient preferences with crossover randomized controlled trials. J Pain Symptom Manag.

[28] Sibbald B, Roberts C (1998). Understanding controlled trials. Crossover Trials BMJ.

[29] Krogh K, Emmanuel A, Perrouin-Verbe B, Korsten MA, Mulcahey MJ, Biering-Sørensen F (2017). International spinal cord injury bowel function basic data set (version 2.0). Spinal Cord.

[30] Krogh K, Perkash I, Stiens SA, Biering-Sorensen F (2009). International bowel function basic spinal cord injury data set. Spinal Cord.

[31] Christensen P, Bazzocchi G, Coggrave M, Abel R, Hulting C, Krogh K (2008). Outcome of transanal irrigation for bowel dysfunction in patients with spinal cord injury. J Spinal Cord Med.

[32] Krogh K, Perkash I, Stiens SA, Biering-Sorensen F (2008). International bowel function extended spinal cord injury data set. Spinal Cord.

[33] Juul T, Bazzocchi G, Coggrave M, Johannesen IL, Hansen RBM, Thiyagarajan C (2011). Reliability of the international spinal cord injury bowel function basic and extended data sets. Spinal Cord.

[34] Mauri L, D’Agostino RB (2017). Challenges in the design and interpretation of noninferiority trials. N. Engl J Med.

[35] Gayet-Ageron A, Agoritsas T, Rudaz S, Courvoisier D, Perneger T (2015). The choice of the noninferiority margin in clinical trials was driven by baseline risk, type of primary outcome, and benefits of new treatment. J Clin Epidemiol.

