# Supplementary material for: Are micro enemas administered with a squeeze tube and a 5 cm-long nozzle as good or better than micro enemas administered with a 10 cm-long catheter attached to a syringe in people with a recent spinal cord injury? A non-inferiority, crossover randomised controlled trial
Source: Spinal Cord. 2022 Jul 27;60(12):1136–43. doi: 10.1038/s41393-022-00835-5 (PMC9328624; doi:10.1038/s41393-022-00835-5)

Kelly LC., Glinsky JV., Garrett G., Nier L., & Harvey LA. Are micro enemas administered with a squeeze tube and a 5cm-long nozzle as good or better than micro enemas administered with a 10cm-long catheter attached to a syringe in people with a recent spinal cord injury? A non-inferiority, crossover randomised controlled trial. 2021

Supplementary File 1: Post hoc analysis with the removal of the two participants who did not adhere to the trial protocol

Table 4. Outcomes by Phases

	**Phase One**		**Phase Two**	
	**Treatment Sequence 1 (squeeze-tube) n = 7**	**Treatment Sequence 2 (catheter) n = 10)**	**Treatment Sequence 1 (catheter) n = 7**	**Treatment Sequence 2 (squeeze-tube) n = 10**
	**$\bar{x}$ (SD)**	**$\bar{x}$ (SD)**	**$\bar{x}$ (SD)**	**$\bar{x}$ (SD)**
**Time to complete bowel care (assessor reported) (mins) Ŧ**	18.2 (6.5)	17.9 (7.5)	16.4 (4.1)	17.3 (5.4)
**Faecal incontinence score system (points/120) Ŧ**	38.9 (33.1)	51.8 (29.4)	29.2 (36.5)	52.1 (39.9)
**SCI-QOL-Bowel Management Difficulties T score (39.2 – 76.3) Ŧ**	52.8 (5.1)	51.5 (6.9)	50.5 (6.7)	52.1 (9.2)
**Perception of effectiveness of bowel care routine (clinician reported) (points 0-10) †**	8.1 (1.1)	7.6 (1.8)	8.0 (0.8)	6.4 (2.4)
**Perception of effectiveness of bowel care routine *(participant reported)* (points 0-10) †**	8.0 (1.8)	8.0 (1.4)	8.2 (1.1)	7.3 (2.6)
**Time to complete bowel care (participant reported) (mins) Ŧ**	17.7 (5.8)	17.1 (6.2)	16.8 (4.9)	17.1 (5.7)

Ŧ A lower score indicates a better outcome

† A higher score indicates a better outcome

Table 5. Outcomes by the two methods of delivering the micro enemas with mean between group difference (95% CI)

	Squeeze-tube method<br>n=17<br>$\bar{x}$ **(SD)**	catheter method<br>n=17<br>$\bar{x}$ **(SD)**	Mean between group difference (95% CI)
**Time to complete bowel care (assessor reported) (mins) Ŧ**	17.6 (5.7)	17.3 (6.2)	-0.4 (-2.8 to 2.1)
**Faecal incontinence score system (points/120) Ŧ**	46.6 (36.7)	42.5 (33.4)	-4.1 (-29.5 to 21.2)
**SCI-QOL-Bowel Management Difficulties T score (39.2 – 76.3) Ŧ**	52.4 (7.6)	51.1 (6.6)	-1.3 (-6.1 to 3.4)
**Perception of effectiveness of bowel care routine (clinician reported) (points 0-10) †**	7.1 (2.1)	7.8 (1.5)	-0.7 (-2.0 to 0.6)
**Perception of effectiveness of bowel care routine (participant reported) (points 0-10) †**	7.6 (2.3)	8.1 (1.2)	-0.5 (-1.8 to 0.8)
**Time to complete bowel care (participant-reported) (mins) Ŧ**	17.3 (5.6)	17.0 (5.5)	-0.3 (-1.8 to 1.2)

Ŧ A lower score indicates a better outcome and a negative between-group difference favours catheter method

† A higher score indicates a better outcome and a negative between-group difference favours squeeze-tube method

Kelly LC., Glinsky JV., Garrett G., Nier L., & Harvey LA. Are micro enemas administered with a squeeze tube and a 5cm-long nozzle as good or better than micro enemas administered with a 10cm-long catheter attached to a syringe in people with a recent spinal cord injury? A non-inferiority, crossover randomised controlled trial. 2021

Figure 6. Mean (95% CI) between group difference for each outcome

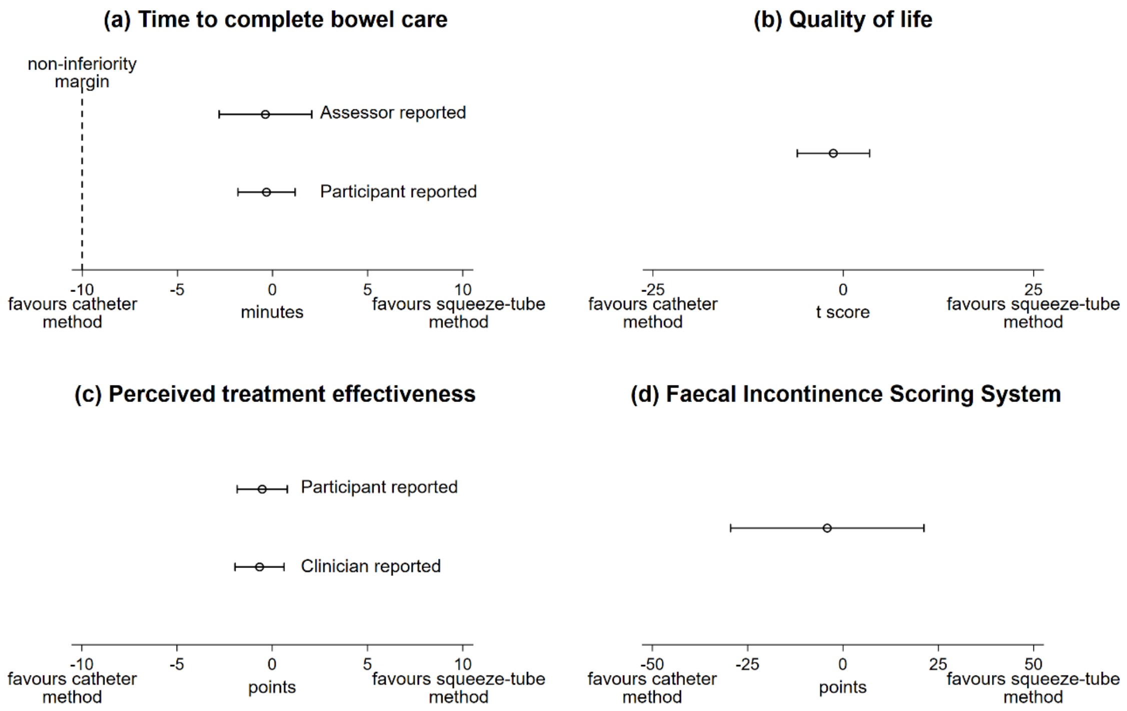

Supplement: Supplementary file 1 — Supplementary File [file 41393_2022_835_MOESM1_ESM.pdf]
